# Yearly Assessment of Bone Disease in Patients with Asymptomatic Multiple Myeloma Identifies Early Progression Events and Should Be the Standard Clinical Practice

**DOI:** 10.3390/jcm14072224

**Published:** 2025-03-25

**Authors:** Ioannis Ntanasis-Stathopoulos, Vassilis Koutoulidis, Panagiotis Malandrakis, Despina Fotiou, Vasiliki Spiliopoulou, Charalampos Filippatos, Magdalini Migkou, Nikolaos Kanellias, Foteini Theodorakakou, Evangelos Eleutherakis-Papaiakovou, Efstathios Kastritis, Evangelos Terpos, Meletios-Athanasios Dimopoulos, Lia-Angela Moulopoulos, Maria Gavriatopoulou

**Affiliations:** 1 Department of Clinical Therapeutics, School of Medicine, General Alexandra Hospital, National and Kapodistrian University of Athens, 11528 Athens, Greece; johnntanasis@med.uoa.gr (I.N.-S.); panosmalandrakis@gmail.com (P.M.); desfotiou@gmail.com (D.F.); vaniaspil@yahoo.com (V.S.); harry.filippatos@gmail.com (C.F.); mmigou@gmail.com (M.M.); nick.kanellias@gmail.com (N.K.); foteint@gmail.com (F.T.); mdeleutherakis@gmail.com (E.E.-P.); ekastritis@gmail.com (E.K.); eterpos@med.uoa.gr (E.T.); mdimop@med.uoa.gr (M.-A.D.); 2 1st Department of Radiology, School of Medicine, Aretaieion Hospital, National and Kapodistrian University of Athens, 11528 Athens, Greece; vkoutoulidis@med.uoa.gr (V.K.); lmoulop@med.uoa.gr (L.-A.M.); 3 Department of Medicine, Korea University, Seoul 02841, Republic of Korea

**Keywords:** smoldering multiple myeloma, multiple myeloma, whole-body low-dose computed tomography, bone, early diagnosis, treatment

## Abstract

Smoldering multiple myeloma (SMM) represents an intermediate stage between monoclonal gammopathy of undetermined significance and symptomatic multiple myeloma (MM), with a significant risk of progression. Bone disease is a key feature of MM, often marking the transition to symptomatic disease. Whole-body low-dose computed tomography (WBLDCT) is an easily accessible and highly sensitive imaging modality for detecting osteolytic lesions, providing an advantage over conventional skeletal surveys. In our real-world cohort, we prospectively evaluated the role of WBLDCT in the early identification of bone progression in patients with SMM based on the recommendations by the International Myeloma Working Group. A total of 113 patients were monitored with annual WBLDCT assessments; 36.3% progressed to symptomatic MM, with 9.7% progressing solely with bone lesions, highlighting the importance of early detection. Therefore, integrating annual WBLDCT assessments into clinical practice for SMM patients is essential to facilitate treatment strategies and prevent disease-related complications. This is even more important in the upcoming era of early treatment initiation for patients with SMM at high risk for progression.

## 1. Introduction

Multiple myeloma (MM) is the second most prevalent hematological malignancy and is characterized by the infiltration of monoclonal plasma cells in the bone marrow and associated end-organ damage [1]. Smoldering multiple myeloma (SMM) is a clinically asymptomatic condition that represents an intermediate stage between monoclonal gammopathy of undetermined significance (MGUS) and multiple myeloma (MM). SMM carries a significantly higher risk of progression to symptomatic MM than MGUS, with an estimated annual progression rate of 10% [2].

The diagnosis of MM is based on the International Myeloma Working Group (IMWG) criteria, which require either bone marrow plasma cell infiltration of ≥10% with at least one CRAB feature (hypercalcemia, renal failure, anemia, or bone lesions) or ≥60% monoclonal plasma cell infiltration in the bone marrow, a serum involved-to-uninvolved free light chain (FLC) ratio of ≥100, more than one focal lesion > 5 mm on magnetic resonance imaging, or a biopsy-proven bone or extramedullary plasmacytoma [3].

Bone disease is the most common symptom associated with MM, affecting more than 80% of patients at some point during the course of their illness [4]. Initial imaging evaluation and monitoring of patients is essential in discriminating the asymptomatic from symptomatic MM [5]. Whole-body low-dose computed tomography (WBLDCT) has been found to be the most sensitive modality for identifying small osteolytic lesions < 5 mm [6,7,8].

## 2. The Role of WBLDCT in Symptomatic MM

WBLDCT is a sensitive technique that provides valuable information for myeloma monitoring, is available in all MM-specialized centers, and has a straightforward approach for imaging without requiring preparation [9,10]. The technique is easy to adopt without the need of additional equipment for imaging laboratories with a CT scanner, and the learning curve for the evaluation and interpretation of the images is short. WBLDCT offers significant advantages as a first-line imaging modality for assessing bone involvement in plasma cell neoplasms, demonstrating a considerably higher sensitivity compared to conventional skeletal surveys in detecting osteolytic lesions, which can justify the initiation of therapy in otherwise asymptomatic patients [11]. A systematic review on 32 directly comparative studies on different methods of imaging in MM patients proved that WBCT and magnetic resonance imaging (MRI) had equal sensitivity and detection rates of osteolytic bone lesions, supporting the implementation of WBCT as standard practice [5].

Systematic evaluation of bone disease—including osteolytic lesions, bone marrow involvement, and fractures—is essential, underlining the importance of sequential imaging. In untreated patients, typical imaging findings include lytic bone lesions larger than 5 mm, which are characterized by a non-sclerotic rim, endosteal scalloping, cortical disruption, pathological fractures, and extraosseous soft tissue masses. Paraskeletal disease and long bone involvement have been associated with a higher tumor burden, advanced disease stage, and poorer prognosis [12]. In a study of 76 patients with active myeloma who underwent assessment with WBLDCT and spinal MRI, 30 (39.5%) had a normal MRI pattern, 15 (19.7%) had a focal MRI pattern, and 30 (39.5%) had a diffuse MRI pattern. In the subset of patients with a diffuse MRI pattern that did not have any lytic lesions (n = 30), WBLDCT was able to identify a diffuse pattern of attenuation in the medullary cavities of the femurs and humeri among 24 individuals (80%), which seems to be associated with advanced disease at diagnosis [13]. All patients with a diffuse pattern of attenuation on WBLDCT had a diffuse pattern on MRI. Therefore, WBLDCT may be enough in terms of diagnostic value for this subset of patients.

In another cohort of 116 patients with plasma cell dyscrasias, including both asymptomatic cases with MGUS or SMM and symptomatic cases with MM, assessment with WBLDCT was proven valuable, as it enabled better patient management. Specifically, 3 patients with plasmacytoma were found to have MM-related bone disease, whereas WBLDCT revealed disease progression in 13 patients, requiring a change in treatment. In addition, in other two MM patients, a previously undiagnosed hepatocellular carcinoma and a rib lesion were detected by WBLDCT, which were subsequently treated by systemic treatment and cryoablation for pain control, respectively [14]. Actually, WBLDCT has a less well-defined role in monitoring patients with MM after treatment completion or during continuous treatment. In clinical practice, WBLDCT assessment is mainly driven by the presence of symptoms that may necessitate further evaluation with imaging. However, ^18^F-FDG-PET/CT or whole body magnetic resonance imaging (WBMRI) may be more suitable in this setting, in order to determine metabolic response to treatment, response of extramedullary disease, unravel early signs of progression, and even aid to treatment decisions such as de-intensification of treatment [12,15,16].

Whole-body diffusion-weighted imaging MRI (WB-DWI-MRI) is becoming more significant in the diagnosis, staging, and evaluation of treatment response in multiple myeloma. In comparison to traditional MRI, WB-DWI-MRI exhibits enhanced sensitivity (86%) and intermediate specificity (63%) for disease identification [17]

In comparison to ^18^F-FDG-PET/CT, WB-DWI-MRI demonstrates superior sensitivity in identifying focal lesions and paraskeletal and diffuse disease across almost all anatomical areas, with the exception of the skull, ribs, scapulae, and clavicles. Furthermore, WB-DWI-MRI has comparable sensitivity in identifying extramedullary disease [18,19,20,21]. The iTIMM study validated the superiority of WB-DWI-MRI compared to ^18^F-FDG-PET/CT, demonstrating greater detection rates of focal lesions (83% against 60%) and diffuse disease (82% versus 17%) [22]. Additional retrospective and prospective investigations have corroborated similar results; nevertheless, the implementation of WB-DWI-MRI in addition to ^18^F-FDG-PET/CT did not substantially impact treatment choices [23]. Moreover, the British recommendations for imaging (MY-RADS criteria) favor the use of WB-DWI-MRI and underscore its prognostic significance in evaluating therapy response [24]. However, it should be noted that WBLDCT remains the first imaging option for the diagnosis of myeloma bone disease according to the current IMWG recommendations, whereas ^18^F-FDG-PET/CT is an alternative. WBMRI is recommended at least for those patients without evidence of myeloma bone disease on WBCT [25]. In a global perspective, it should be also underlined that WBCT is an easily accessible imaging technique, associated with less logistics, costs, and need for specialized equipment, facilities, and personnel, as well as patient discomfort, as compared to WBMRI or even a PET/CT scan.

## 3. The Role of WBLDCT Assessment in the Asymptomatic Precursors of MM

### 3.1. Findings from Previously Published Prospective and Retrospective Studies

Early identification of disease progression via thorough active monitoring allows for risk-adapted treatment strategies that may delay or even prevent end-organ damage in SMM patients. The recently published, randomized, and phase 3 AQUILA trial allocated 390 high-risk SMM patients to treatment with daratumumab or active monitoring. In both patient groups, surveillance for evolution to symptomatic disease consisted of repetitive comprehensive assessments until confirmation of disease progression. This is especially important for cases with a biochemical increase in paraprotein or involved free light chain levels, which are considered a major risk factors for progression. In this pivotal study, treating patients with high-risk SMM with subcutaneous daratumumab for 3 years was associated with a significantly lower risk of progression to active multiple myeloma or death, as well as a trend for improved overall survival compared to active monitoring [26].

Furthermore, the importance of timely identification of progression to symptomatic disease has been shown in previous studies. The IMWG conducted a retrospective study in order to compare WBCT with conventional skeletal survey (CSS), enrolling a total of 212 individuals with myeloma. Among them, 66 were diagnosed with SMM and 54 with newly diagnosed symptomatic MM. A total of 44 cases were discordant with WBCT, detecting osteolyses that were invisible with CSS. Among patients with disease classified as SMM based on standard X-rays, 12 (22.2%) had osteolytic lesions identified via WBCT, and they had to be reclassified as symptomatic MM, according to the current standards [27]. In another small study, assessment with WBLDCT in 25 patients with MGUS and 15 patients with SMM was able to detect MM-related bone-disease in 14 of them [10 with MGUS (10/25, 40%) and 4 with SMM (4/25, 27%)], leading to a change of management [13].

### 3.2. Updated Findings from a Prospective Evaluation of Asymptomatic MM Patients Based on Yearly WBLDCT Assessments

Herein, we provide an update of our previous study assessing the role of WBLDCT in the early identification of patients with SMM who progress solely with bone disease and require immediate antimyeloma therapy, with a longer follow-up period and an extended cohort [28]. We evaluated prospectively 113 patients (13 new patients, 13% larger cohort) with asymptomatic MM who underwent WBLDCT assessments at baseline, 1 year post diagnosis and every 1 year thereafter. The patients enrolled in the study were those who had at least two consecutive CT assessments at the above-described time points and were followed with hematologic, biochemical, and immunological tests every 3 months for the first two years, and every 6 months thereafter. Eligibility criteria and methods of assessment, evaluation, and analysis have been described previously [28].

The median age at diagnosis for our cohort was 60 years (range 35–85 years) and 53.1% were females. The median number of WBLDCT exams conducted was three, with a range of 2–6. According to the IMWG 2/20/20 risk stratification model, 36.3% of the patients were classified as low risk, 33.6% as low-intermediate risk, 24.0% as intermediate risk, and 4.4% as high risk (Table 1).

During a median follow-up period of 8.81 years (IQR 7.3–10.7 years), 41 patients (36.3%) progressed, according to the CRAB-SLiM criteria. Notably, 11 of these (26.8% out of progressors and 9.7% of the whole cohort) progressed solely with bone lesions that were detected on WBLDCT. Those patients were clinically asymptomatic and had no other SLiM criteria or signs of biochemical progression, as determined by the IMWG criteria for disease progression in patients with symptomatic myeloma. Furthermore, one patient had inconclusive results on WBLDCT regarding the presence of osteolytic lesions and required a WBMRI that revealed a normal pattern to exclude symptomatic myeloma. The median follow-up period was 8.82 years for bone-only progressors compared to 10.05 years for other progressors. Within the subgroup of bone-only progressors, 9% were in the high-risk stage, 46% were in the intermediate-risk stage, 36% were in the low-intermediate stage, and 9% were in the low-risk stage.

No significant differences were observed between patients with bone-only progression and those with other forms of progression regarding baseline hemoglobin, albumin, β2-microglobulin, free light chains (FLCs), bone marrow infiltration, and other baseline characteristics (Wilcoxon, *p* > 0.05 for all). Among the 30 other progressors, progression was associated with anemia (n = 6), bone marrow infiltration greater than 60% (n = 3), and abnormal free light chain ratio exceeding 100 (n = 3), and 18 patients experienced more than one CRAB/SLiM event (11 with two criteria, 5 with three, and 2 with four).

**Figure 1:**
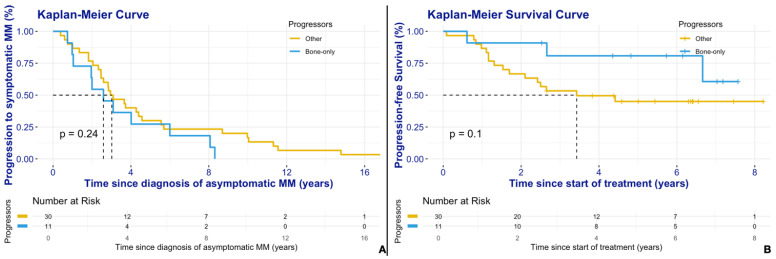
Kaplan–Meier curves depicting (**A**) the time to progression (TTP) from smoldering to symptomatic MM for bone-only progressors and other progressors, and (**B**) the progression-free survival (PFS) among patients with evolution to symptomatic multiple myeloma (n = 41) for bone-only progressors and other progressors.

The median time to progression (TTP) from asymptomatic to symptomatic disease for all 113 patients was not reached (NR). For those who actually progressed, the median TTP was 2.95 years (95% CI: 2.46, 4.39). In the subgroup of patients who progressed with bone lesions only, the median TTP was 2.59 years (95% CI: 1.96, NA), compared to 3.02 years (95% CI: 1.96, NA) for other progressors. There was no statistically significant difference between the two progressor subgroups (Figure 1A, *p* = 0.244). Moreover, the number of lytic lesions (more than 10, compared to less than 10) identified by WBLDCT among bone-only progressors, was not related to TTP (HR = 0.76, 95% CI: 0.17–3.73, *p* = 0.736).

At the time of progression to symptomatic disease, bone marrow infiltration had significantly increased from baseline in patients with bone-only progression (44.4% vs 25.5%, *p* = 0.028). The distribution of patients per ISS stage at the time of progression was as follows: 56.1% in stage 1, 36.6% in stage 2, and 7.3% in stage 3 for all progressors. For those who progressed only with bone lesions, the distribution was 72.7% in stage 1 and 27.3% in stage 2. The R-ISS stage distribution for all progressors was 46.3% in stage 1, 43.9% in stage 2, and 9.8% in stage 3. For bone-only progressors, the distribution was 63.6% in stage 1 and 36.4% in stage 2. There were no significant differences in ISS and R-ISS stage distributions between the two subgroups.

All patients began anti-myeloma treatment immediately after diagnosis of symptomatic disease. A total of 19 patients experienced disease progression after first-line treatment: 3 (27.3%) from the bone-only subgroup and 16 (53.3%) from the others. The median progression-free survival (PFS) for the 41 patients with symptomatic MM was 6.66 years (2.66, NA). For bone-only progressors, the median PFS was not reached, and for other progressors, it was 3.43 years (2.10, NA). There was no statistically significant difference between the two subgroups (Figure 1B, *p* = 0.113). Furthermore, the number of lytic lesions (more than 10, compared to less than 10) identified by WBLDCT among bone-only progressors was not associated with PFS (HR = 2.32, 95% CI: 0.20–26.6, *p* = 0.500).

Overall, there were three deaths: two among the patients who progressed to MM (one MM-related death during the third line of treatment, one not related to MM) and one among the non-progressors (not related to SMM). None of the patients who died had progressed with isolated bone involvement.

## 4. Key Take-Away and Conclusions

Although yearly WBLDCT examinations in SMM may be an additional burden for both patients and physicians, herein we highlight the importance of complying with the IMWG recommendations in clinical practice [25]. In patients with SMM and a negative initial assessment with both WBLDCT and WBMRI, yearly follow-up with WBMRI is recommended in order to detect emergent focal lesions, even without underlying osteolysis. However, WBMRI may be impractical in all settings, taking also into consideration that not all patients with SMM are followed in myeloma expert centers across the globe. Herein, we showed that yearly assessments with WBLDCT may be a feasible alternative, especially given the lack of prospective studies directly comparing sequential WBLDCT and WBMRI in patients with SMM. Among our patients, 36.3% progressed to symptomatic MM, and in roughly 10% of the cases, progression was identified solely by the presence of bone lesions on WBLDCT without other CRAB criteria or myeloma-defining events. Moreover, a numerical trend of better PFS outcomes among bone-only progressors compared to others, in our cohort, underlines the potential benefit of timely diagnosis (avoiding the potential risks and comorbidities associated with a late diagnosis) and early treatment intervention. However, the results were not statistically significant; therefore, larger studies with longer follow-up periods are required to validate this observation. Furthermore, it would be valuable to investigate the role of a tailored approach regarding the frequency of imaging studies according to the risk stratification of progression to symptomatic disease. This could be a dynamic approach by evaluating prospectively the risk by the 20-20-2 IMWG model or the Pangea models in successive time points and adjusting the imaging surveillance accordingly [29,30,31]. Supplemental imaging may be needed for patients showing rapidly increasing biochemical parameters such as those fulfilling the IMWG MM criteria for biochemical progression, but this has to be validated in prospective studies.

Overall, these findings underscore the utility of WBLDCT in detecting early bone disease, enabling timely therapeutic interventions and potentially preventing end organ damage due to delayed diagnosis. This is even more relevant when taking into consideration the results of the Aquila study [26], as well as a meta-analysis of 21 clinical trials suggesting that early intervention in patients with high-risk SMM significantly reduces the risk of progression to symptomatic MM and mortality [32].

In conclusion, the evidence presented underscores the crucial role of early detection of disease progression among SMM patients and highlights WBLDCT as a valuable imaging modality in this setting. Given the strong association between radiologically detected bone lesions and progression risk, incorporating yearly WBLDCT assessments into the standard practice of SMM care can help refine patient monitoring and management.

## Figures and Tables

**Table 1:** Baseline patient characteristics.

Variable	All Patients (n = 113)	Bone-Only Progressors (n = 11)	Other Progressors (n = 30)	*p*-Value
Age (years)	60 (35–85)	58 (52–72)	60.5 (38–83)	0.385
Females (%)	53.1	45.5	60	0.498
WBCT (n)	3 (2–6)	2 (2–4)	3 (2–4)	0.069
Hb (g/dL)	12.9 (8.2–16.0)	13.7 (11.4–14.8)	12.6 (9.5–14.7)	0.510
Cr (mg/dL)	0.8 (0.4–8.3)	0.8 (0.5–1.3)	0.71 (0.4–1.5)	0.361
Ca (mg/dL)	9.4 (5.4–11.1)	9.4 (8.5–10.6)	9.6 (5.4–10.6)	0.350
B2 microglobulin (mg/L)	2.2 (0.9–15.1)	2.2 (1.1–4.1)	2.3 (0.9–4.0)	0.672
LDH (U/L)	169 (103–325)	158 (103–221)	168 (109–274)	0.804
Alb (g/dL)	4.3 (3.2–5.3)	4.1 (3.7–4.8)	4.2 (3.3–4.8)	0.907
IgG (mg/dL)	1550 (358–5824)	1940 (626–4170)	1720 (420–5824)	0.758
IgA (mg/dL)	109.5 (5–4181)	50.9 (14–1336)	103 (22–1650)	0.758
IgM (mg/dL)	43.5 (4.2–369)	36.4 (17.4–171)	40.0 (4.2–205.0)	0.596
M-peak (g/dL)	1.5 (0.0–4.9)	2.6 (0.9–3.7)	1.8 (0.0–4.9)	0.382
κFLC (mg/L)	19.5 (1.3–990.0)	26.8 (11.5–635.0)	25.0 (1.3–990.0)	0.638
λFLC (mg/L)	12.4 (1.1–988.0)	11.8 (5.2–760.0)	10.1 (1.1–988)	0.369
FLC ratio > 8 (n, %)	30 (26.5)	5 (45.5)	14 (46.7)	0.617
BM infiltration (%)	20 (2.4–55.0)	20 (10–40)	27.5 (10–55)	0.666
Heavy chain (n) IgG IgA	82 28	7 4	7 22	
Light chain (n) kappa lambda	1 2	0 0	1 0	-
Progression risk (%) ^a^ Low Low-Intermediate Intermediate High	36.3 33.6 24.0 4.4	9.1 36.4 45.5 9.1	13.3 30.0 46.7 10.0	0.987

Notes: Values are expressed as median (range). Significance *p*-values are for bone versus other and come from Chi-square, Fisher’s exact, and Mann–Whitney tests where appropriate. ^a^: According to the IMWG 2/20/20 risk stratification model.

## Data Availability

The original contributions presented in this study are included in the article. Further inquiries can be directed to the corresponding author.
